# Levetiracetam Improves Upper Limb Spasticity in a Patient With Unresponsive Wakefulness Syndrome: A Case Report

**DOI:** 10.3389/fnins.2020.00070

**Published:** 2020-02-06

**Authors:** Valeria Pingue, Marta Gentili, Anna Losurdo, Emilio Clementi, Antonio Nardone

**Affiliations:** ^1^Neurorehabilitation and Spinal Unit, Institute of Pavia, Istituti Clinici Scientifici Maugeri IRCCS, Pavia, Italy; ^2^Pharmacy Service, Institute of Pavia, Istituti Clinici Scientifici Maugeri IRCCS, Pavia, Italy; ^3^Unit of Clinical Pharmacology, Department of Biomedical and Clinical Sciences L. Sacco, University Hospital “Luigi Sacco”, Università di Milano, Milan, Italy; ^4^Scientific Institute IRCCS Eugenio Medea, Lecco, Italy; ^5^Department of Clinical-Surgical, Diagnostic and Pediatric Sciences, University of Pavia, Pavia, Italy

**Keywords:** pain, disorders of consciousness, hypoxic-encephalopathy, unresponsive wakefulness syndrome, spasticity, levetiracetam

## Abstract

Severe spasticity is a frequent and disabling complication in patients presenting disorders of consciousness (DOC) that hinders their rehabilitative process, and is strongly correlated with pain reducing patients’ quality of life. In these patients, abnormal postures may occur as an expression of severe brain damage. Here we present the case of a 52-year-old man in decorticate rigidity following a hypoxic–ischemic encephalopathy due to myocardial infarction who showed improvement of spasticity of upper limbs following intake of levetiracetam combined with the conventional neurorehabilitation program.

## Background

The improvements in post-resuscitation care over the recent decades have significantly given rise to an increase in patients presenting disorders of consciousness (DOC). DOC includes patients in coma, in vegetative/unresponsive wakefulness syndrome and in minimally conscious state (Jennett and Plum, 1972; Multi-Society Task Force on PVS, 1994; Giacino et al., 2002).

Up to 89% of patients with DOC suffer from spasticity characterized by atypical clinical patterns related to widespread lesions at various levels of the central nervous system and poorly controlled by the standard pharmacological treatments (Thibaut et al., 2015b; Martens et al., 2017). While this syndrome and its management are well-known in patients suffering from stroke, multiple sclerosis or spinal cord lesion, there are no guidelines regarding its appropriate management in DOC patients (Martens et al., 2017). In this population, spasticity has particularly negative impacts (e.g., muscle contractures, loss of range of movement, bedsores) and is strongly correlated with pain (Thibaut et al., 2015b). In addition, spasticity involves long-term complications and difficulties in nursing activities that consistently reduce patients’ quality of life and possibility of functional recovery.

## Case Presentation

In January 2018, a 52-year-old man in a prolonged vegetative state was admitted to the Neurorehabilitation Unit of the Istituti Clinici Scientifici Maugeri of Pavia, following a hypoxic– ischemic encephalopathy due to cardiac arrest that had occurred in November 2017. On admission, the patient’s Glasgow Coma Scale (GCS) total score was 9/15 (eye opening: 4; verbal response: 1; motor response: 4), the Nociception Coma Scale-Revised (NCS-r) was 1/9 (motor response: 0; verbal response: 0; facial expression: 1). Consciousness level assessed with the Coma Recovery Scale-Revised (CRS-r) was 3/23 (auditory: 0; visual: 1; motor: 0; oromotor: 1; communication: 0; arousal: 1).

From the history, the patient was a smoker (about 40 cigarettes a day from his youth), suffered from type 2 diabetes mellitus and hypertension; before the cardiac arrest, he never showed any neurological symptoms.

Already at the admission to our Unit chance of improvement in DOC was very poor in this patient, considering that predictors of unfavorable outcome can be even defined few days after cardiac arrest (Oddo and Rossetti, 2011; Taccone et al., 2014; Paul and Legriel, 2019). Magnetic Resonance Imaging of the brain showed bilateral damage to the cerebral hemispheres, internal capsule, and basal ganglia. Evaluation of median nerve somatosensory evoked potentials reported severe abnormalities (absent N20 and bilaterally prolonged N9). The patient never presented symptomatic seizures or recordings of electroencephalographic interictal epileptiform discharges.

Already at the admission in our Unit (5 weeks after cardiac arrest), the subject presented decorticate rigidity as a consequence of the severity of brain damage: shoulders adducted, elbows flexed, legs and feet extended. The initial evaluation of spasticity, assessed with the Modified Ashworth Scale (MAS), was severe especially in the upper limbs, where MAS scored bilaterally four for shoulders and three for wrists and elbows. In the lower limbs, MAS scored bilaterally three for hips, knees and ankles. According to clinical and instrumental findings, the primary and realistic neurorehabilitation goals were reduction of spasticity. Physical therapy included postural exercises, mobilization and stretching of all four limbs. Intrathecal baclofen therapy was contraindicated due to the high risk of bleeding since the patient was being administered double antiplatelet therapy (acetylsalicylic acid 100 mg/day and clopidogrel 75 mg/day). Therefore, antispasmodic treatment (baclofen 100 mg/day and clonazepam 6 mg/day) was administered via the oral route. In addition, EMG-guided Botulinum Toxin-A (abobotulinumtoxinA) injections were performed to treat the upper limbs according to the following scheme: 250 U in right and left pectoral; 250 U in right and left biceps; 80 U in right and left pronator quadratus.

An improvement of spasticity was observed after 2 months in particular for the lower limbs, where MAS value for hips and knees bilaterally reduced to two, thus allowing hygienic maneuvers and sitting in a wheelchair for several hours during the day. On the contrary, the improvements in the upper limbs were poor: in particular, the elbows remained in a flexed position allowing only a 10-degree passive extension bilaterally, the wrists were flexed at 90° and the shoulders adducted with a MAS score equal to 4. The severity of spasticity made nursing procedures and mobilization difficult and associated with signs of pain. Three months after admission to our Institute, the patient, who had never showed symptomatic seizures or recordings of electroencephalographic interictal epileptiform discharges, presented a seizure related to a feverish episode. Levetiracetam (LEV; 2000 mg/day) was then added. In the following days, an improvement in spasticity of the upper limbs was observed by the physiotherapist who was treating the patient on routinely administration of the MAS: indeed, its score reduced to three for shoulders and two for elbows and wrists. This improvement was confirmed on administration of the MAS in the following days although the patient remained in vegetative/unresponsive wakefulness syndrome (CRS-r total score was 4/23). In particular, the elbow passive extension range of motion (pROM) increased by 50° and these improvements occurred bilaterally simplifying patient’s nursing care and daily treatment. The pROM improvement allowed splinting the upper limbs, the latter being considered a further efficient option to reduce hand spasticity in patients with DOC (Thibaut et al., 2015a).

**FIGURE 1:**
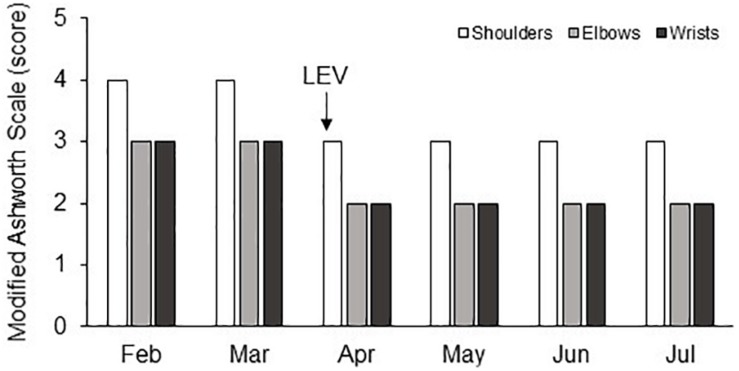
Changes in the MAS score (vertical axis) in shoulder, elbow, and wrist joints of both upper limbs during the 6 months of hospitalization (horizontal axis).

The patient’s condition appeared stable during the following hospital stay. On discharge (July 2018), the GCS total score was 9/15 (eye opening: 4; verbal response: 1; motor response: 4), the NCS-r was 2/9 (motor response: 1; verbal response: 0; facial expression: 1), and the CRS-r was 4/23 (auditory: 1; visual: 1; motor: 0; oromotor: 1; communication: 0; arousal: 1). The improvement in spasticity of the upper limbs was still persisting without further changes of the oral administered antispastic and antiepileptic therapy (Figure 1).

## Discussion

The peculiarity of this case consists in the unexpected improvement of spasticity resulting from post-anoxic encephalopathy following the administration of LEV in spite of a lack of improvement with the previous usual therapy based on baclofen, benzodiazepines and botulinum injections.

Levetiracetam is a second-generation antiepileptic drug approved in 1990 mainly employed in the treatment of epilepsy in patients with DOC. Several studies have proved that it also possesses neuroprotective effect (Shetty, 2013; Sterkel et al., 2017) and low potential for interaction with other medications and for side effects. The mechanism of action of LEV has not yet been fully elucidated to date but differs structurally and functionally from that of the other available anti-epileptic drugs.

There is evidence in the literature that LEV not only inhibits neuronal firing discharge but also decreases muscle spasticity in patients with neurodegenerative diseases (Bedlack et al., 2009) and phasic (but not tonic) spasticity in patients with multiple sclerosis (Hawker et al., 2003).

Based on the mechanism of action of LEV, it is possible to hypothesize the way through which it would have led to an improvement in spasticity. LEV plays a role in Ca^2+^ homeostasis: it is able to inhibit both inositol triphosphate (IP3) and ryanodine-dependent Ca^2+^ release from the endoplasmic reticulum. Furthermore, LEV blocks the L-type Ca^2+^ channels in the hippocampal neurons, as well as those present in the smooth and striated muscles, inhibiting Ca^2+^ entry and its intracellular increase (Micov et al., 2010). LEV also acts on the N type Ca^2+^ channels, and it is able to reduce the release of glutamate by acting on the P/Q voltage-dependent channel (Chun-Yao et al., 2009). Besides the effect on ion channel activity, LEV is able to modulate GABA receptors at the cortical and spinal level (Solinas et al., 2008). Such mechanisms would lead to the reduction of neurotransmission and muscle contractility, thus explaining why LEV may lead to an overall improvement of spasticity.

Interestingly, LEV may have had a potential anti-hyperalgesic effect in our patient although we did not find changes of the NCS-r. Several studies have suggested that an anti-hyperalgesic effect could be modulated by several receptors: alpha2-adrenoreceptors, GABAA receptors, opioids and serotonin 5-HT1, which is present at the dorsal horn level, in the spinal cord and gray matter. The activation of serotonin 5-HT1, binding to one of its own ligand as LEV, could have a role in the modulation of nociceptive input (Bardin et al., 2000; Micov et al., 2010). In particular, Eide et al. (1990) examined the administration of elevated doses of LEV, 5-HT1A, and 5-HT1B receptor agonists, and found that their activation could be related to the inhibition of the peripheral input, positively modulating spasticity.

It is possible that the combined action of the above two mechanisms (reduction of muscle contractility and modulation of nociceptive input) has been successful where the other pharmacological treatments have failed.

Given the difficulty in determining tolerability in a patient with disturbance of consciousness, we cannot exclude the occurrence of adverse drug reactions across this period although none was clinically evident. We acknowledge that, even in absence of accepted guidelines, treatment with oral baclofen and clonazepam at high doses should be anyhow carefully considered in patients with DOC, due to sedative side effects. However, this drug association is justified when reduction of spasticity and prevention of tertiary damage are the only realistic goals, as in our patient who showed since admission unfavorable clinical and instrumental findings related to recovery of consciousness.

A limitation of this case report is that we have no clinical data of the patient after discharge from our ward, because it was not possible to plan the usual outpatient follow up given the severe disability of the patient. Although LEV likely had an effect on spasticity due to the close temporal relationship between the onset of therapy and the occurrence of improvement, from the available data, we cannot exclude that antispastic effects are from add-on LEV to baclofen, clonazepam and botulinum toxin treatment rather than exclusively from LEV. Finally, also combined effects or even delayed effects from multi-professional management, oral antispastic drugs and focal treatment with botulinum toxin might have a permissive role in the appearance of the antispastic effects of LEV.

## Conclusion

Currently, in DOC there are no guidelines to manage spasticity, and this is certainly connected to the complexity of the mechanisms underlying severe brain injury. Without any further evidence, treatment for spasticity has to be adapted individually and certainly multidisciplinary approach combining physical, pharmacological and surgical treatments is the key to manage spasticity in patients with DOC, keeping the way open for more effective treatments.

Our observation that following LEV administration an improvement of spasticity has been observed in a patient with severe brain injury suggests that LEV may be considered in future guidelines as an adjunct for spasticity treatment in patients with DOC. However, *ad hoc* clinical controlled trials are necessary to verify any positive effect in spasticity in such a selected set of patients.

## Data Availability Statement

The datasets generated for this study are available on request to the corresponding author.

## Ethics Statement

The patient’s wife’s written informed consent was obtained for their participation in the study, in accordance with the Declaration of Helsinki, and for publication of the case report. No investigation or intervention was performed outside routine clinical care for this patient. As this is a case report, without experimental intervention into routine care, no formal research approval is required.

## Author Contributions

VP contributed to the conception and design and wrote the first draft of the manuscript. MG contributed to design and wrote the section “Discussion” of the manuscript. All authors contributed to manuscript revision, and read and approved the submitted version.

## Conflict of Interest

The authors declare that the research was conducted in the absence of any commercial or financial relationships that could be construed as a potential conflict of interest. The handling Editor declared a past co-authorship with one of the authors, EC.
